# Candidate gene networks and blood biomarkers of methamphetamine-associated psychosis: an integrative RNA-sequencing report

**DOI:** 10.1038/tp.2016.67

**Published:** 2016-05-10

**Authors:** M S Breen, A Uhlmann, C M Nday, S J Glatt, M Mitt, A Metsalpu, D J Stein, N Illing

**Affiliations:** 1 Department of Clinical and Experimental Sciences, Faculty of Medicine, University of Southampton, Southampton, UK; 2 Department of Psychiatry and MRC Unit on Anxiety and Stress Disorders, Groote Schuur Hospital (J-2), University of Cape Town, Cape Town, South Africa; 3 Department of Molecular and Cellular Biology, University of Cape Town, Cape Town, South Africa; 4 Psychiatric Genetic Epidemiology and Neurobiology Laboratory, Departments of Psychiatry and Behavioral Sciences and Neuroscience and Physiology, Medical Genetics Research Center, SUNY Upstate Medical University, Syracuse, NY, USA; 5 The Estonian Genome Center, University of Tartu, Tartu, Estonia

## Abstract

The clinical presentation, course and treatment of methamphetamine (METH)-associated psychosis (MAP) are similar to that observed in schizophrenia (SCZ) and subsequently MAP has been hypothesized as a pharmacological and environmental model of SCZ. However, several challenges currently exist in diagnosing MAP accurately at the molecular and neurocognitive level before the MAP model can contribute to the discovery of SCZ biomarkers. We directly assessed subcortical brain structural volumes and clinical parameters of MAP within the framework of an integrative genome-wide RNA-Seq blood transcriptome analysis of subjects diagnosed with MAP (*N=*10), METH dependency without psychosis (MA; *N=*10) and healthy controls (*N=*10). First, we identified discrete groups of co-expressed genes (that is, modules) and tested them for functional annotation and phenotypic relationships to brain structure volumes, life events and psychometric measurements. We discovered one MAP-associated module involved in ubiquitin-mediated proteolysis downregulation, enriched with 61 genes previously found implicated in psychosis and SCZ across independent blood and post-mortem brain studies using convergent functional genomic (CFG) evidence. This module demonstrated significant relationships with brain structure volumes including the anterior corpus callosum (CC) and the nucleus accumbens. Furthermore, a second MAP and psychoticism-associated module involved in circadian clock upregulation was also enriched with 39 CFG genes, further associated with the CC. Subsequently, a machine-learning analysis of differentially expressed genes identified single blood-based biomarkers able to differentiate controls from methamphetamine dependents with 87% accuracy and MAP from MA subjects with 95% accuracy. CFG evidence validated a significant proportion of these putative MAP biomarkers in independent studies including *CLN3*, *FBP1*, *TBC1D2* and *ZNF821* (RNA degradation), *ELK3* and *SINA3* (circadian clock) and *PIGF* and *UHMK1* (ubiquitin-mediated proteolysis). Finally, focusing analysis on brain structure volumes revealed significantly lower bilateral hippocampal volumes in MAP subjects. Overall, these results suggest similar molecular and neurocognitive mechanisms underlying the pathophysiology of psychosis and SCZ regardless of substance abuse and provide preliminary evidence supporting the MAP paradigm as an exemplar for SCZ biomarker discovery.

## Introduction

Methamphetamine (METH) is an *N*-methyl derivative of amphetamine and a highly addictive psychostimulant severely affecting the central nervous system.^1^ METH use is at epidemic levels in several areas of the world and its global prevalence is estimated at 15–16 million people with several pockets of increased use in the USA, Europe and Africa.^2, 3^ Recent evidence ranked METH fourth out of 20 of the most harmful drugs due to self-harm to the user.^4^ One reason for this is that METH provokes psychotic reactions in an estimated 72–100% of all abusers.^5, 6^

Methamphetamine-associated psychosis (MAP) has been considered a pharmacological and environmental model of schizophrenia (SCZ) due to similarities in clinical presentation (that is, paranoia, hallucinations, disorganized speech and negative symptoms), response to treatment (neuroleptics) and presumed neuromechanisms (central dopaminergic neurotransmission).^7, 8, 9^ It is hypothesized that a better understanding of the molecular mechanisms underlying SCZ may be accelerated via examination of human models related to the disease. In this context, the MAP model could quicken the discovery of risk biomarkers, screening for subclinical disease, prognostics, diagnostics or disease staging. However, several challenges currently exist in terms of accurately diagnosing MAP on a molecular and cognitive level before the MAP model can contribute to the discovery of SCZ biomarkers.

Genome-wide blood transcriptome profiling coupled with network analyses provide a platform for identifying functionally relevant biological markers of disease, permitting multi-scale data integration.^10^ This is a critical point as acute and chronic effects of MAP are widespread across the body and an integrative technique determining relationships of biological markers with magnetic resonance imaging (MRI), life events (that is, stress, culture) and psychometric measurements could provide key insights towards cognitive and molecular mechanisms of MAP, and the versatility of the MAP model in molecular psychiatry research. Complimentary, machine learning provides a useful tool for *in silico* prediction of candidate biomarkers.^11^ Further confirmation and validation of these biomarkers may be accomplished by utilizing convergent functional genomics (CFG) evidence. The CFG approach has proven highly successful for moderately sized psychiatric cohorts in reducing false positives and false negatives by drawing on multiple disparate yet ‘convergent' sources of external functional genomic information across independent human studies.^12, 13, 14, 15, 16, 17, 18, 19, 20^ Collectively, these techniques hold great promise for the prioritization and validation of candidate genes for MAP and their relatedness to SCZ.

We present a preliminary integrative RNA-sequencing report exploring peripheral blood gene expression among subjects diagnosed with METH-associated psychosis (MAP), METH dependency without psychotic symptoms (MA) and healthy control subjects. The primary goal of this analysis was to best characterize the molecular signatures defining MAP at the systems level and again at the individual gene level to reveal a novel panel of MAP blood biomarkers. An unbiased weighted gene co-expression network analysis (WGCNA) was first used to identify co-expression modules that were subjected to functional annotation and multi-scale data integration collected from the same subjects. Subsequently, a multi-class machine-learning approach was used to identify candidate blood biomarkers able to differentiate between MA, MAP and healthy control subjects. CFG information was used to validate the role of candidate gene networks and blood biomarkers in the pathophysiology of MAP and confirm their shared association to psychotic disorders and SCZ in independent studies with the absence of METH.

## Materials and methods

### Participants

A total of 10 MAP subjects, 10 subjects with METH dependence without developing psychotic symptoms (MA), and 10 healthy control subjects were enrolled in this study. Gender (male) and age-matched (25.8±6 years) right-handed subjects were recruited from drug rehabilitation facilities, hospitals and communities in Cape Town, South Africa where all the subjects were provided detailed study information and gave written consent. Each subject underwent two assessment sessions. The first session consisted of a detailed psychiatric interview and demographic and substance variables were recorded. During the second session, approximately 1 week later, the patients were asked to fast and refrain from smoking overnight, before blood was collected between 0900 and 1100 h. This was followed by a brain scan. Clinical assessment was performed using the Structured Diagnostic Interview for DSM-IV Axis I Disorders^21^ and the patients completed a battery of self-report questionnaires including the Life Events Questionnaire,^22^ Kessler Psychological Distress Scale (K10),^23^ the Beck Depression Inventory,^24^ behavioural inhibition system/behavioural activation system scale,^25^ Eysenck Personality Questionnaire—Revised short scale^26^ (For detailed information regarding each of these measures, see Supplementary File). Positive and negative symptoms within the MAP group were rated using the PANSS (Positive and Negative Syndrome Scale):^27^ PANSS positive subscale (14.5±6.1), negative subscale (22.0±11.5) and total score (66.8±26.1). Exclusion criteria comprised the following: (1) additional substance dependencies other than nicotine and METH for the MA and MAP groups, and any substance dependence other than nicotine in the control group; (2) lifetime and current diagnosis of any psychiatric disorders (other than MA dependence and MAP in the MA and MAP groups); (3) a history of psychosis before MA abuse; (4) a medical or neurological illness or head trauma; (5) a seropositive test for HIV; (6) MRI incompatibilities or known claustrophobia. All the participants in the MAP group were on treatment with neuroleptic medication (haloperidol) at the time of testing. Polysubstance use was allowed to facilitate participant recruitment including nicotine, cannabis and alcohol for all the study groups. This study was approved (HREC REF 340/2009) by the University of Cape Town Faculty of Health Sciences Human Research Ethics Committee.

### MRI acquisition and image processing

The subjects in this study form part of a larger project investigating fronto-temporal cortical and subcortical grey matter structures in MA and MAP. The images were acquired on a 3 T Magnetom Allegra scanner (Siemens, Erlangen, Germany) at the Cape Universities Brain Imaging Centre. A high-resolution, T1-weighted, three-dimensional multi-echo MPRAGE sequence (scan parameters: repetition time=2530 ms; graded echo time=1.53, 3.21, 4.89, 6.57 ms; flip angle=7° field of view=256 mm) produced 160 sagittal images of 1 mm thickness. By acquiring four separate structural scans with graded echo times and averaging those into a final high contrast image,^28^ the MEMPRAGE method creates structural images with low distortion and high signal-to-noise ratio.

The MRI scans were analysed using the FreeSurfer software package v5.1 (http://surfer.nmr.mgh.harvard.edu/). Regional estimates of subcortical volumes were assessed with a specialized surface-based reconstruction and automatic labelling tool, which is described in detail elsewhere.^29^ In summary, FreeSurfer processing includes motion correction, skull-stripping, Talairach transformation, segmentation of subcortical white matter and deep grey matter volumetric structures, intensity normalization, tessellation of the grey matter/white matter boundary, automated topology correction and surface deformation.

### RNA isolation, library preparation and data availability

Blood was collected using PAXgene RNA tubes (Qiagen, Valencia, CA, USA) and total RNA was extracted and purified in accordance with the PAX gene RNA kit per manufacturer's instructions. Globin mRNA was depleted from samples using the GLOBINclear—Human Kit (Life Technologies, Carlsbad, CA, USA). Subsequently, the quantity of all purified RNA samples was measured on a nanodrop (56.6±16.7 ng μl^−1^) and the quality and integrity measured with the Agilent 2100 Bioanalyzer (Agilent, Santa Clara, CA, USA). All RNA integrity numbers were greater than 7 (8.4±0.7).

The Illumina TruSeq Stranded Total RNA kit (Ilumina, San Diego, CA, USA) was used for library preparation accordingly to manufacturer instructions without any modifications. The 30 indexed RNA libraries were pooled and sequenced using long paired-end chemistry (2x93 bp) on seven lanes using the Illumina HiSeq2500. All the replicates were run for 2 × 40 million reads per sample and all the reads were primary processed using Casava v1.8.2 to transform primary base call files into fastq files. These raw RNA-sequencing fastq data have been submitted to Gene Expression Omnibus (http://www.ncbi.nlm.nih.gov/geo/) under accession number GSE74737.

### Read trimming, mapping and quantification of gene expression

All the fragmented RNA-Seq reads were trimmed to 90 bp and low quality reads were discarded using Trimmomatic^30^ options SLIDINGWINDOW:90:10 MINLEN:90 CROP:90. Subsequently, all high-quality trimmed reads were mapped to UCSC *Homo sapiens* reference genome (build hg19) using TopHat v2.0.0.^31^ We used the estimated mean inner distance and standard deviation between mate paired-ends as the -r and —mate-std-dev parameters, respectively. TopHat calls Bowtie v1.1.1^32^ to perform alignment with no more than two mismatches. We used the pre-built index files of UCSC *H. sapiens* hg19, downloaded from the TopHat homepage (https://ccb.jhu.edu/software/tophat/igenomes.shtml). Samtools^33^ was used to convert bamfiles to samfiles and HTseq v0.6.0^34^ was used to count all of the mapped reads by htseq-count using parameters –stranded=reverse –q.

### Data pre-processing

Raw count data measured 23 345 transcripts across 30 subjects. Unspecific filtering removed lowly expressed genes that did not meet the requirement of a minimum of 20 reads in at least 10 subjects. A total of 12 281 transcripts were retained, then subjected to edgeR VOOM normalization,^35^ a variance-stabilization transformation method. Normalized data were inspected for outlying samples using unsupervised hierarchical clustering of subjects (based on Pearson coefficient and average distance metric) and principal component analysis to identify potential outliers outside two standard deviations from these averages. No outliers were present in these data and resulting normalized values were used as input for downstream analyses.

### Gene co-expression network construction and module detection

Signed co-expression networks were built using WGCNA^10^ in R, as previously described.^36, 37^ A total of 12 281 transcripts were used to construct a global network of all 30 subjects. To construct a network, the absolute values of Pearson correlation coefficients were calculated for all the possible gene pairs and resulting values were transformed using a β-power of 9 so that the final correlation matrix followed an approximate scale-free topology.^10^ The WGCNA cut-tree hybrid algorithm was used to detect sub-networks, or co-expression modules, within the global network optimizing minimum module size to 15, deep split of 2 and a tree-cut height of 0.2 to merge neighbouring network modules with similar expression profiles. For each identified module, we ran singular value decomposition of each module's expression matrix and used the resulting module eigengene (ME), equivalent to the first principal component, to represent the overall expression profiles for each module. Differential expression of MEs was performed using a Bayes analysis of variance^38^ (parameters: conf=12, bayes=1, winSize=5) testing between groups and *P*-values were corrected for multiple comparisons (*post hoc* Tukey correction). Subsequently, to determine which modules were most associated to recorded clinical parameters and potential confounding variables in this study, MEs for all modules were correlated to external subjective and objective data using a Pearson correlation and a Student's asymptotic *P*-value for significance. MEs were also used to determine module membership (kME) values for each gene in a specified module, defined as the correlation between gene expression values and ME expression. Genes with the highest intramodular kME were labelled as hub genes and predicted to be essential to the function of the module.

### Differential gene expression analyses

A moderated *t*-test, implemented through the *limma*^39^ package, assessed differential gene expression between the three groups in a group-wise manner across 12 281 transcripts. Significance threshold was set to a nominal *P*-value <0.01 to permit sufficient enough genes to move forward with functional characterization and supervised classification methods. Differentially expressed genes corresponding to WGCNA modules which were significantly associated with polysubstance abuse were excluded and removed from functional annotation and supervised classification methods, as a robust and complimentary strategy of adjusting for confounding factors.

### Functional enrichment analyses

All differentially expressed genes passing a *P*-value <0.01 and all network modules with genes passing a kME>0.50 were subjected to functional annotation. First, the ToppFunn module of ToppGene Suite software^40^ (https://toppgene.cchmc.org/) was used to assess enrichment of GO ontology terms relevant to cellular components, molecular factors, biological processes, metabolic pathways and well-annotated drug compounds from the comparative toxicogenomics database^41^ using a one-tailed hyper-geometric distribution with a Bonferroni correction. A minimum of a two-gene overlap per gene-set was necessary to be allowed for testing. The human cell-specific gene expression database from the cell type enrichment^42^ analysis web-based tool was used to predict the involvement of key cell types within candidate gene lists. For each supplied gene list, the significance of cell type-specific expression are determined using the one-tailed Fisher's exact test with a Bonferroni correction across all the available cell/tissue types. For information pertaining to curating haloperidol gene signatures, see Supplementary File.

### Construction of diagnostic blood classifier for MAP

BRB-Array Tools^11^-supervised classification methods were used to construct gene expression classifiers. Two models were specified: (1) controls vs METH dependents and (2) MA vs MAP subjects. Each model consisted of three steps. First, to ensure a fair comparison and to decrease computational time, all genes with *P*<0.01 were subjected to classifier construction. This heuristic rule of thumb approach was used to cast a wide net to catch all potentially informative genes, while false positives would be pared off by subsequent optimization and cross-validation steps. Second, classifiers composed of different numbers of genes were constructed by recursive feature elimination. Recursive feature elimination provided feature selection, model fitting and performance evaluation via identifying the optimal number of features with maximum predictive accuracy. Third, the ability for recursive feature elimination to predict group outcome was assessed by diagonal linear discriminant analysis and compared with three different multivariate classification methods (that is, support vector machine, nearest centroid, three-nearest neighbours) in a leave-one-out cross-validation approach. In addition, a permutation *P*-value, based on 1000 random permutations, for the cross-validated misclassification error rate for each classification method was implemented. This *P*-value indicates the proportion of the random permutations that gave as small a cross-validated misclassification rate as was obtained with the real class labels.

### Converging functional genomic scoring

CFG represents a translational methodology that integrates multiple lines of external evidence from human and animal model studies in a Bayesian-like manner. This approach increases the ability to distinguish signal from noise in limited size cohorts and is routinely applied to support the identification of blood biomarkers across neuropsychiatric disorders.^12, 13, 14, 15, 16, 17, 18, 19, 20^ The principal aim of the CFG approach is to increase the likelihood that findings will prove reproducible and have predictive power in independent cohorts. Our CFG scoring paradigm for prioritization of MAP biomarkers is an adaptation of previous techniques, representing a two-step process (Supplementary Figure 6) as given below.

*Internal lines of evidence*: All genes assigned a *P*-value <0.05 were included in the CFG scoring. These liberal criteria were used to cast a wide net of all potentially informative genes, which may be involved in the pathophysiology of MAP, while false positives would be pared off by subsequent CFG scoring and optimization steps. Each gene was given three *P*-values (based on three group-wise differential expression analyses). Subsequently, a score of 1 was given to genes passing *P*<0.001, a score of 0.5 was given to genes passing 0.001>*P*<0.01, and a score of 0.2 was given for genes passing 0.01>*P*<0.05, permitting a maximum score of 3 and a minimum score of 0.2. A bonus point of 0.5 was awarded for genes passing *P*<0.01 occurring in both MAP vs controls and MAP vs MA comparisons, as well as genes found to be members of MAP-associated modules. Thus, a max score of 4 is attainable (3+0.5+0.5).

*External lines of evidence*: CFG evidence was scored for a gene if there were published reports of human data including post-mortem brain expression, peripheral blood expression and/or genetic evidence (association and linkage) utilizing two large databases. One database represents a recently built in-house database specific to human blood transcriptome studies using PubMed (http://www.ncbi.nlm.nih.gov/pubmed) search queries and combinations of key words (e.g. blood transcriptome and psychosis).^43^ To consider functional support across divergent technological platforms and human post-mortem brain samples, we accessed DisGenNet,^44^ a comprehensive database of human gene–disease associations from various expert curated databases and text-mining-derived associations. These database searches included gene–disease relationships focusing specifically on psychosis, SCZ, depression/stress and neurocognitive impairment to consider comorbid effects of MAP in our study. Importantly, studies containing a METH component were excluded in order to validate MAP biomarkers in drug-free (METH) models. For the CFG analysis and scoring, external cross-validating lines of evidence were weighted such that findings in human peripheral blood specific to psychosis were given an additional 1 point. A maximum of five external lines of evidence were allowed. Thus, the total maximum CFG score that a candidate biomarker gene could have was 10 (4 for threshold+5 for external evidence+1 blood presence in psychosis). Like other studies using this approach,^12, 13, 14, 15, 16, 17, 18, 19, 20^ we appreciate there are other ways of scoring blood biomarkers based on CFG which may give slightly different results in terms of prioritization.^12, 13, 14, 15, 16, 17, 18, 19, 20^ Given the past utility of this approach, we and others believe that this empirical scoring system allows for advantageous separation of genes based on our focus for identifying human MAP blood biomarker and by default, biomarkers of psychosis and SCZ.

## Results

**Figure 1:**
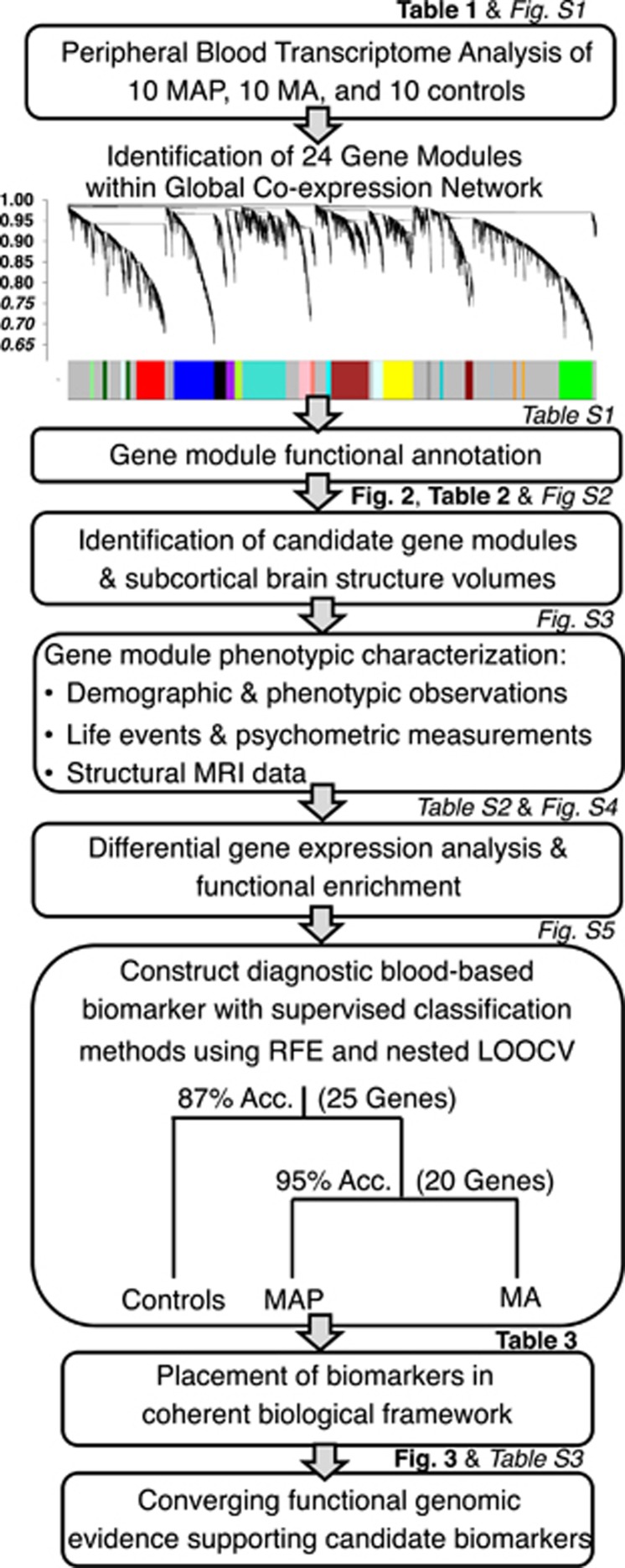
A multi-step translational work-flow for identifying methamphetamine-associated psychosis (MAP) biomarkers. First, weighted gene co-expression network analysis (WGCNA) analysis built a global co-expression network and identified 24 co-expression modules. On the hierarchical cluster tree, each line represents a gene (leaf) and each group of lines represents a discrete group of co-regulated genes or gene modules (branch) on the clustering gene tree. Each gene module is indicated by the colour bar below the dendrogram, and subsequently functionally annotated then integrated with recorded clinical and biological data to identify candidate gene modules representing functional biomarkers of MAP. Second, differential gene expression and class prediction methods identified 20 candidate MAP biomarkers (14 were recycled from the second split on the tree). A Bayesian-like convergent functional genomic (CFG) approach prioritized our panel of biomarkers specific to MAP and biomarkers were placed within an empirically derived biological framework. For each step, the corresponding figure and/or table is listed providing a quick reference. LOOCV, leave-one-out cross-validation; RFE, recursive feature elimination.

We conducted a preliminary integrative RNA-sequencing study profiling peripheral blood gene expression from a primary cohort of 10 MA, 10 MAP and 10 healthy controls (Table 1 and Supplementary Figure 1). To identify and prioritize diagnostic blood biomarkers of MAP, a multimodal translational approach was used (Figure 1). A global gene co-expression network was first constructed using all the available subjects and identified 24 co-expression modules, which were functionally annotated to molecular factors, biological processes, cellular compartments, metabolic pathways, well-characterized drug compounds and cell type specificity (Supplementary Table 1).

### Differential analysis of ME values and brain structure volumes

**Figure 2:**
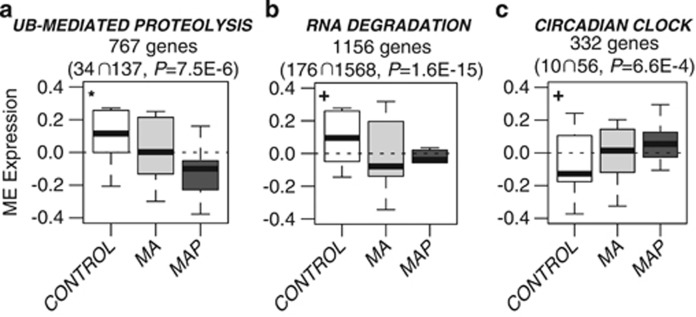
Significant methamphetamine-associated psychosis (MAP) findings from differential analysis of module eigengene (ME) values across controls (white), MA subjects (light grey) and MAP subjects (dark grey). Modules specific to MAP include (**a**) ubiquitin (UB)-mediated proteolysis, (**b**) RNA degradation and (**c**) circadian clock. Indicated for each module are number of overlapping genes from the module ∩ out of total genes in the term. Enrichment *P*-values are Bonferroni corrected for multiple comparisons. A Bayes analysis of variance (parameters: conf=12, bayes=1, winSize=5) was used on the ME values to test for significance between the groups and *P*-values were corrected for multiple comparisons where (*) implies *post hoc*-corrected *P*-value significance <0.05 and (^+^) indicates *P*-value significance <0.05 without *post hoc* correction.

To reduce the number of multiple testing corrections and false positives arising from standard differential gene expression analyses, we calculated differences in module expression using ME values (See Materials and methods for complete description of ME). All the ME values were subjected to a Bayes analysis of variance^32^ testing to compare the extent of module expression between the groups and the *P*-values were corrected for multiple comparisons. MAP-associated findings included significant decreases of ME expression in modules specific to ‘ubiquitin-mediated proteolysis' (767 genes) and ‘RNA degradation' (1156 genes) in MAP subjects compared with controls (*P*=0.01, *P*=0.03, respectively; Figures 2a and b). Further, an increase of ME expression in a module annotated as ‘circadian clock' (332 genes) was observed in MAP compared with controls (*P*=0.04; Figure 2c). MA-associated findings included the increase of ME expression in modules specific to ‘chloride transporter activity' (106 genes), ‘interferon signalling' (263 genes) and ‘cytokine signalling' (186 genes), and a decrease of ME expression in modules associated to ‘generic transcription' (48 genes) and ‘ribosome pathway' (281 genes) in MA subjects relative to healthy controls (Supplementary Figure 2). The same methodology was extended to compare the brain structural volumes (mm^3^) across the three groups, which revealed bilaterally reduced hippocampus volumes in MAP subjects (left, *P*=0.04; right, *P*=0.02; Table 2).

### Phenotypic characterization of MAP modules

The ME values for MAP-specific modules were correlated with all phenotypic traits in this study (brain structural volumes, life history and psychometric measures) to gain insight into the role that each module may have in the pathophysiology of the disorder (Supplementary Figure 3). The *P*-values <0.002 pass the most conservative multiple comparison correction (Bonferroni). The ME of a ‘ubiquitin-mediated proteolysis' module was negatively associated to MAP status (*r*=−0.45, *P*=0.01) as well as K10 total score (*r*=−0.43, *P*=0.02). Interestingly, this module was also negatively associated with brain structure volumes in areas of the anterior CC (*r*=−0.55, *P*=0.002), right accumbens area (*r*=−0.40, *P*=0.03) and positively associated to areas in the left caudate (*r*=0.37, *P*=0.04) and left ventral diencephalon (DC, *r*=0.48, *P*=0.007). The ‘RNA degradation' module was negatively associated with the CC anterior (*r*=−0.48, *P*=0.008) and left accumbens (*r*=0.50, *P*=0.005), while positively associated with the left ventral DC (*r*=0.37, *P*=0.04). The ‘circadian clock' module, was positively correlated with EPQRS measure of psychoticism (*r*=0.43, *P*=0.02) and negatively associated to extraversion (*r*=−0.36, *P*=0.04).

### Phenotypic characterization of MA modules

A similar strategy was chosen to characterize MA-specific modules (Supplementary Figure 3). The ME of the ‘interferon signalling' module was positively associated to MA status (*r*=0.40, *P*=0.03), BDI total score (*r*=0.40, *P*=0.03), as well as structural information from both left (*r*=0.54, *P*=0.002) and right putamen areas (*r*=0.41, *P*=0.03). This module was negatively associated to EPQRS measure of extraversion (*r*=−0.38, *P*=0.04) and EPQRS total score (*r*=−0.38, *P*=0.04). Further, the ME of the ‘chloride transporter activity' module was positively associated with both MA status (*r*=0.36, *P*=0.05) and METH dependency (*r*=0.39, *P*=0.03), in addition to BDI total score (*r*=0.39, *P*=0.03) and brain volume in the left putamen (*r*=0.53, *P*=0.003). This module was also negatively associated to control status (*r*=−0.39, *P*=0.03) and the left ventral DC (*r*=−0.40, *P*=0.03). The ‘ribosome pathway' module was negatively associated to MA status (*r*=−0.37, *P*=0.04) and positively associated to EPQRS total score (*r*=0.38, *P*=0.04) and K10 total score (*r*=0.44, *P*=0.02). The ‘cytokine signalling' module was positively associated with both left accumbens (*r*=0.37, *P*=0.04) and right accumbens (*r*=0.55, *P*=0.002), whereas the ‘generic transcription' module was negatively associated to these areas (*r*=−0.49, *P*=0.006; *r*=−0.60, *P*=5e-04, respectively).

### Putative diagnostic blood biomarker for MAP

Supervised class prediction methods were used to identify any single important gene(s) that may have been over-looked in our network analysis. First, differentially expressed genes (all *P*<0.01) were identified between the control and MA subjects (*N=*197), control and MAP subjects (*N=*409) and between the MA and MAP subjects (*N=*79; Supplementary Table 2, Supplementary Figures 4A–D). To control for confounding factors, genes corresponding to WGCNA modules significantly associated to polysubstance abuse were excluded. Gene lists were annotated for functionality at the pathway level and cross-referenced with drug-induced gene signatures from the comparative toxicogenomics database (Supplementary Figure 4E and F; See Supplementary File for detailed information).

Subsequently, differentially expressed genes (*P*<0.01) were pooled from across the three candidate gene lists and subjected to recursive feature elimination feature selection and different multivariate classification methods in a leave-one-out cross-validation approach (See Materials and Methods for complete description). Two models were built for separating classes. First, when separating healthy controls form METH dependents (MA and MAP subjects) classification accuracy reached 87% when the expression of 25 genes was used with diagonal linear discriminant analysis multivariate classification method (Supplementary Figures 5a and b). Second, when separating MA from MAP, classification accuracy reached 95% when the expression of 20 genes (recycling 14 genes from the first model) was used with diagonal linear discriminant analysis (Supplementary Figures 5c and d).

We next sought to understand the biology represented by these MAP biomarkers and derive mechanistic insights. Our multi-step approach permitted taking each single biomarker and returning to our network analysis to retrieve guilt-by-association biological information from our empirically derived functional gene modules. Majority of these genes were found in a module annotated to ‘RNA degradation' (*CLN3*, *FBP1*, *TBC1D2*, *ZNF821*, *ADAM15*, *ARL6*, *FBN1* and *MTHFSD*; Table 3). However, two top-scoring biomarkers were found to be implicated in ‘circadian clock' dysfunction (*ELK3* and *SINA3*) and three other top-scoring biomarkers were found in the module annotated to ‘ubiquitin-mediated proteolysis' (*PIGF*,*UHMK1* and *C7orf11*).

### Prioritization and biological interpretation of blood biomarkers

Biomarkers were prioritized using a Bayesian-like CFG approach (Supplementary Figure 6) integrating previously published human evidence based on genetics (for example, GWAS, copy number variants), post-mortem brain gene expression and peripheral blood gene expression specific to psychosis, SCZ, depression/stress as well as neurocognitive impairment at the time of our analysis (August 2015). This is a way of validating relevant blood transcriptome biomarkers from moderately sized data sets, extracting generalizable signal out of potential cohort-specific noise.^12, 13, 14, 15, 16, 17, 18, 19, 20^ Using the CFG approach, we first focused our attention on the ‘ubiquitin-mediated proteolysis' annotated module, which in this study represents a functional biomarker of MAP. This module was enriched with 61 genes having CFG evidence (*P*=4.8E−10), including those found to be dysregulated in the blood of a psychotic disorder (*n*=29) as well as in the blood and/or post-mortem brain of SCZ patients (*n*=32) across independent human studies (Supplementary Table 3A). Notably, of the 29 CFG genes found in the blood of a psychotic disorder, 21 pertained to one single study.^45^ We further found a significant enrichment of 39 genes holding CFG evidence (*P*=7.0E−12) within the module annotated as ‘circadian clock' (Supplementary Table 3B). Similarly, these genes were also previously associated to psychosis and/or SCZ in independent studies. Of interest, two genes within the ‘ubiquitin-mediated proteolysis' annotated module (*TMEM106B* and *SCAMP1*) and one within the ‘circadian clock' annotated module (*DCTN1*) overlap with a previous study that had used CFG-based approach to validate blood biomarkers for delusions, a core symptom of psychotic disorders.^20^ An additional gene (*RAB18*) within the ‘ubiquitin-mediated proteolysis' module was also validated as a SCZ biomarker using the CFG approach.^18^

**Figure 3:**
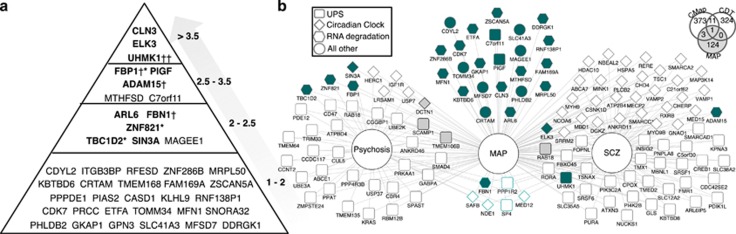
Top candidate blood biomarkers for methamphetamine-associated psychosis (MAP). (**a**) Convergent functional genomic (CFG) evidence and scoring are depicted on the right side of the pyramid. Genes in bold have been found in external publications. Genes found in methamphetamine (METH)-free studies investigating schizophrenia (SCZ, ^†^) and psychosis (*) are as indicated. (**b**) Overlapping gene–disease relationships including CFG-validated genes within gene modules (ubiquitin-mediated proteolysis and circadian rhythm) and single-gene biomarkers. Nodes represent genes and edges indicate gene–disease relationships. Node shape denotes empirically derived functions from our network analysis. Green shading indicates biomarkers from our machine-learning analysis including 14 unique genes separating controls from METH dependants. Grey nodes represent CFG-validated biomarkers of delusion (psychosis) or SCZ.^12, 18^ Node border colour in turquoise indicates gene signatures across MAP, general psychosis and SCZ studies. Venn diagram depicts lack of overlap from curated haloperidol gene signatures onto the 128 candidate MAP genes (61 UPS+39 clock+25+20=128 genes (while accounting for overlap across lists)).

Applying the CFG approach to our panel of 31 discriminative biomarkers confirmed 8 candidate biomarkers for MAP (Table 3) which had a CFG score of 3 or above, meaning either maximal score from the *P-*value threshold cut-offs or at least two other lines of prior independent evidence (Figure 3a). Indeed, CFG evidence for 8 out of 31 discriminatory biomarkers is a significant overlap (*P*=0.01), beyond what would be expected by chance. Of these validated MAP biomarkers, four were previously reported to predict psychosis in an independent human blood transcriptome investigation (*FBP1*, *ZNF821*, *TBC1D2* and *SIN3A*), one of which was previously labelled a genetic variant for SCZ risk (*FBP1*). In addition, one other biomarker had been implicated in SCZ risk across two independent studies (*UHMK1*). Subsequently, a gene–disease network was built using all the CFG-validated biomarkers, either in the form of a functional biomarker (gene modules) or single biomarkers, to visualize unique gene signatures of MAP and consensus signatures of MAP, psychosis and SCZ (Figure 3b). In this study, we found that MAP shares 69 genes with SCZ, 39 genes with other psychotic disorders and six genes are shared across all the three conditions. Importantly, cross-referencing all the candidate MAP genes onto query haloperidol gene expression signatures from the CMap and CDT provided preliminary evidence for the lack of neuroleptic-associations across our candidate findings (Figure 3b).

## Discussion

This preliminary report describes gene networks and blood biomarkers of MAP, further validating the MAP model as an exemplar for discovery of biomarkers related to SCZ susceptibility and clinical course. In essence, this pharmacogenomics approach is a tool for identifying genes that contain pathophysiological relevance to psychotic disorders and SCZ. Considering the variable environmental component of MAP, it is possible that not all subjects would show changes in all the biomarker genes. Hence, our multimodal approach incorporated blood gene expression, clinical assessment of life history, psychometric measures and structural MRI data revealing several mechanistic insights regarding the pathophysiology of MAP and its overlapping mechanistic nature with psychotic disorders and SCZ. First, we identified a functional biomarker of MAP in the form of a co-expression module annotated to ubiquitin-mediated proteolysis, further enriched with 61 genes containing CFG evidence. We also revealed a psychoticism-associated module implicated in circadian clock, enriched with 39 genes containing CFG evidence. Second, we identified 25 genes that were able to distinguish healthy controls from METH dependents with high accuracy, while only 20 genes (recycling 14 genes from the previous split) were able to differentiate between MA and MAP subjects. A significant proportion of these single blood biomarkers also contained CFG evidence. Further, cross-referencing these results onto haloperidol specific gene expression signatures reduced the likelihood of these genes being neuroleptic-related. These high overlaps suggest similar biological mechanisms detectable in peripheral blood underlying the pathophysiology of psychosis, regardless of substance abuse. These findings also outline new avenues regarding how the MAP model may function in SCZ research.

A central finding from our network analysis was the identification of a functional biomarker (gene module) annotated to ubiquitin-mediated proteolysis expressed to a lesser extent in MAP subjects (Figure 2a). The ubiquitin proteasome system (UPS) is a highly complex and tightly regulated process that has major roles in a variety of basic cellular processes, specifically degradation of intracellular proteins and modulation of cellular responses to inflammation and oxidative stress.^46^ The UPS has been identified in genetic reports as a canonical pathway associated to psychosis,^45, 47^ SCZ,^48, 49, 50, 51, 52^ bipolar disorder,^48, 53^ as well as neurodegenerative diseases such as Alzheimer's^54^ and Parkinson's.^55^ Studies using post-mortem brain gene expression to investigate mechanisms of psychosis and SCZ provide consistent evidence for the downregulation of UPS-related genes in these conditions.^50, 51, 52^ It was also recently shown that UPS abnormalities disrupt expression at the protein level in SCZ.^56^ Interestingly, studies using peripheral blood gene expression also found that the UPS pathway was consistently dysregulated across bipolar, SCZ and psychosis patient groups.^48^ A later study used a targeted approach associating blood expression measurements of UPS pathway gene members with Scales for Assessment of Positive and Negative Symptoms and determined *UBE2K* (also a gene member of our ‘ubiquitin-mediated proteolysis' module), was one of three genes most significantly associated to positive symptoms of psychosis.^47^ Another independent report built a diagnostic blood-based classifier able to distinguish first-episode psychosis from controls with 400 genes,^45^ 21 of which were found within our UPS annotated module (Supplementary Table 3A). Indeed, it is interesting that genes that have a well-established role in brain functioning should also show changes in peripheral blood in relationship to psychiatric symptom states, and moreover that the direction of change should be concordant with that reported in human post-mortem brain studies. As a consequence of the overlapping nature of UPS dysfunction found across mental diseases, the proteasome system has emerged as a putative candidate highlighting both mRNA and protein-level changes in psychosis and SCZ. This clearly is an area that deserves attention and mechanistic elucidation by future hypothesis-driven research.

In determining relationships between blood gene expression and structural MRI data, we revealed a significant association of the ubiquitin-mediated proteolysis module to the anterior CC (*r*=−0.55, *P*=0.002; Supplementary Figure 3). Conversely, the circadian clock module, expressed to a greater extent in MAP subjects (Figure 2), was significantly associated to EPQRS measure of psychoticism (that is, aggression, egocentrism and impulsiveness; *r*=0.43, *P*=0.02) and the posterior CC (*r*=0.39, *P*=0.03; Supplementary Figure 3). There is considerable evidence suggesting that global white matter abnormalities (that is, disruptions in connectivity in intra- and interhemispheric pathways) have a role in the pathophysiology of psychiatric disorders.^57^ With the CC being the largest white matter tract containing highly packed neuronal fibres, abnormalities in this structure have frequently been reported in patients with SCZ,^58^ including first-episode SCZ and psychosis patients,^59^ often relating to the severity of psychotic symptoms. It has been hypothesized that less efficient connectivity and resulting aberrant signal transmission between the brain regions may be a pivotal factor in the manifestation of psychotic symptoms, including delusions and hallucinations, and of cognitive dysfunctions.^60, 61^ However, these disturbances have not been fully elucidated in the context of MAP nor in its relationship to blood gene expression differences. Yet most interestingly, we also observed significantly lower bilateral hippocampal volumes in MAP subjects (Table 2). Although correlates of blood gene expression to hippocampal volumes relate mainly to processes of protein ubiquitination (*r*=0.37, *P*=0.05), reductions in the hippocampal volumes are consistent with previous reports of pathological hippocampus changes in MAP,^62^ in first-episode and chronic schizophrenia,^63^ and in individuals at high risk for psychosis.^64^ Taken together, these results suggest that changes in the blood occur in parallel to structural changes in the brain of MAP subjects and that they are also most likely involved in the pathophysiology of psychotic disorders and SCZ in the absence of METH.

The interrogation of the comparative toxicogenomics database^41^ with a signature query composed of the genes in our ‘ubiquitin-mediated proteolysis' annotated module revealed an enrichment of sodium arsenate gene signatures (Supplementary Table 1). Although sodium arsenate is one of the most toxic metals derived from the natural environment,^65^ it has been used as a therapeutic medication in acute promylocytic leukaemia based on its mechanism to induce apoptotic effects via release of apoptosis-inducing factor.^65^ However, arsenic is mainly a contaminator and interestingly is known to cause clinical features such as psychosis, toxic cardiomyopathy and seizures.^66,67^ This exploratory result suggests arsenic, and chemically similar compounds, as a putatively useful gene-hunting tool for investigating future mechanisms of psychosis in either primary or patient-derived lymphoblast cell lines to elucidate further these effects in search for more verifiable biomarkers.

Topping our list of candidate MAP biomarkers, we found eight genes involved in RNA degradation (*CLN3*, *FBP1*, *TBC1D2*, *ZNF821*, *ADAM15*, *ARL6*, *FBN1* and *MTHFSD*), two specific to circadian rhythm (*ELK3* and *SINA3*) and three involved in ubiquitin-mediated proteolysis (*PIGF*,*UHMK1* and *C7orf11*; Table 3). Indeed it is possible that some of the gene expression changes detected in this moderately sized cohort (*N=*30) may represent biological or technical artefacts. To minimize such effects, our candidate MAP biomarkers were selected based on having a line of evidence (CFG) score of two or higher (Figure 3a). Proper cross-validation both *in silico* and across-literature (CFG), minimized the likelihood of having identified false positives while increasing sensitivity and specificity in the ability to distinguish true signal (biomarkers) from noise through a fit-to-disease Bayesian-like methodology.^12, 13, 14, 15, 16, 17, 18, 19, 20^

*CLN3* (Ceroid-Lipofuscinosis, Neuronal 3) was the top-scoring gene in our study and is conventionally involved in lysosome function. Mutations in this gene are well known to cause neurodegenerative diseases such as Batten disease,^66, 68^ which impairs mental and motor development during childhood, causing difficulty with walking, speaking and intellectual functioning. Patients with a CLN3 mutation are also prone to recurrent seizures, epilepsies, vision impairment and occasionally psychosis. It is hypothesized that mutations in CLN3 disrupt lysosome function resulting in build-up of lipopigments, which may induce apoptotic effects in brain neurons. Although this gene has not yet been discussed in the context of psychosis, it may represent a putative biomarker of MAP. In addition, variants in the gene *FBP1* (fructose-1,6-bisphosphatase 1) have previously provided genetic support for the view that alterations in glucose metabolism are intrinsic to SCZ pathology.^69^ However, in our study, this gene was found co-expressed in the ‘RNA degradation' module. Other top-scoring genes included genes annotated to a circadian clock module (Supplementary Table 3B), which are involved in sleep–wake cycles and previously identified as risk factors for psychosis,^12^ anxiety disorders,^17^ suicidality^19^ and mood disorders.^70^ *ELK3* (ETS-Domain Protein (SRF Accessory Protein 2)) encodes a transcriptional factor that may switch from activator to repressor in the presence of Ras, whereas *SIN3A* (SIN3 Transcription Regulator Family Member A) encodes a transcriptional repressor with known roles in circadian clock negative feedback.^71^ Although *SIN3A* has well-known association to circadian clock function, an advantage of our approach was to be able to derive guilt-by-association co-expression interpretation of biomarkers, such as *ELK3*, by indicating module membership status. Dysregulation of circadian clock genes in post-mortem brain of SCZ patients have previously been observed,^72^ however, reports in the blood are less frequent.

Of note, MA-associated findings also allow us to speculate on molecular mechanisms of psychosis. MA discoveries mainly included elevated expression in modules specific to interferon and cytokine signalling. Although cytokine signalling was positively associated to METH dependency (that is, MA and MAP subjects; *r*=0.39, *P*=0.03), a module specific to ‘interferon signalling' was significantly overexpressed in the blood of MA subjects relative to controls, rather than MAP subjects relative to controls (Supplementary Figure 2). Previous work has highlighted a weak or absent immune stress response, specific to HPA axis activation^73^ and cortisol measurements,^74^ in medication-naive first-onset psychosis patients. Moreover, modules specific to IL-5 signalling, actin cytoskeleton and ATPase activity all showed a strong association to both the left and right accumbens area (Supplementary Figure 3). Owing to high levels of dopaminergic innervations, the nucleus accumbens, together with other subcortical structures, has a pivotal role in several neurocircuits involved in reward, motivation, drug-reinforcement and drug-seeking behaviour, mood regulation and sleep–wake cycles.^75, 76^ Such neurocircuit functions are similarly affected by drug exposure as well as stressors, life events or social pressure, with increased dopamine release in the nucleus accumbens triggered by the stimulant in addiction and by glucocorticoid hormones in stress.^75^ Furthermore, there is emerging evidence that cytokines circulating in blood may target subcortical dopamine function, with potential implications on behaviour, sleep patterns and the progression of psychiatric disorders, such as depression.^77^

Although it appears that the identification of blood-based biomarkers may be accomplished by systems level and machine-learning approaches, it remains an open empirical question for future work, which approach provides the most favourable translational avenues. Systems approaches are particularly useful in providing comprehensive characterizations of the molecular factors for a given disease state, multi-scale data integration and are statistically robust in terms of reproducibility. Machine-learning applications, while often fit-to-cohort, rank genes by importance producing a unique predictive or diagnostic panel of biomarkers. This dual approach permitted the placement of MAP single-gene biomarkers into an empirically derived biological framework (that is, gene network) to derive mechanistic insights. Pragmatically, these results provide a proof of principle for joint statistical analysis providing complimentary and comprehensive molecular characterizations in pursuit of blood biomarkers for MAP. A limitation of this study is that our findings cannot yet be used to change the clinical practice. Notwithstanding that many of our MAP single-gene biomarkers identified by machine learning were supported by CFG evidence, these findings need to be replicated in an independent MAP sample.

Overall, our results support the MAP model for the identification of biomarkers involved in psychosis and SCZ. Our most significant findings suggest that genes involved in UPS and circadian clock dysregulation are prominent players in psychosis and are reflected in both peripheral blood and post-mortem brain profiles. Specifically, UPS abnormalities have emerged as a common denominator across a variety of independent studies investigating psychosis, SCZ and bipolar disorder. Indeed in clinical practice there is a high degree of overlap and comorbidity between psychotic disorders, MAP and SCZ. Our results were able to shed light on the biological mechanisms of psychosis, regardless of polysubstance abuse, medication or other confounding factors and further emphasize the value of moving towards comprehensive empirical profiling. These results also open empirical avenues for future field trials, clinical testing and validation in various at-risk populations.

## Figures and Tables

**Table 1:** Recorded clinical characteristics from all subjects (*N*=30)

	*Healthy controls* *(*N*=10)*	*MA* *(*N*=10)*	*MAP* *(*N*=10)*	*ANOVA*		Post hoc *significance*
	*Mean±s.d.*	*Mean±s.d.*	*Mean±s.d.*	X^*2*^*(df=2)*	P*-value*	*Bonferroni* P*-value*
Age	25.5±5.8	24.8±3.9	27.2±8.3	0.040	0.980	
Education level	12.2±1.2	10.7±2.1	9.3±1.7	10.788	0.005	Contol > MAP
METH age started using	—	18.6±3.9	18.8±6.8	0.191	0.662	
METH abstinence (days)	—	53.1±82.9	45.5±36.2	0.593	0.441	
METH duration of use (years)	—	5.8±2.3	7.1±3.0	0.688	0.407	
Nicotene use last 30 days	5	6	9	2.400	0.121	
Cannabis use last 30 days	2	2	1	0.529	0.467	
Alcohol use last 30 days	3	4	2	1.347	0.246	
EPQRS psychoticism	2.3±1.7	1.6±1.2	3±2.1	1.880	0.391	
EPQRS extraversion	10.3±2.5	8.2±3.5	6.6±2.5	7.039	0.030	Contol > MAP
EPQRS neuroticism	2.6±1.8	4.6±2.9	5.6±3.2	4.624	0.099	
EPQRS lie	5.6±2.3	4±1.9	5.1±3.3	1.902	0.386	
EPQRS total score	20.8±5.3	18.5±2.3	20.4±4.7	1.876	0.391	
BIS	15.1±1.5	15.8±3.1	13.1±3.6	3.018	0.221	
BAS drive	7.4±2.5	8.3±2.6	6.5±1.3	2.267	0.322	
BAS fun seeking	7.1±1.5	8.1±1.6	6±1.2	7.014	0.030	MA > MAP
BAS reward responsiveness	7.7±1.9	7.2±1.8	6.2±1.7	3.859	0.145	
BIS/BAS total score	44.8±5.8	47±7.9	38.4±5.6	6.269	0.044	
BDI total score	4.3±3.0	17.3±10.3	16.6±12.5	10.363	0.006	MAP > Control; MA > Control
K10 total score	14±3.8	18.2±7.7	23.5±8.2	7.944	0.019	MAP > Control
LEQ—sum of life events (⩽6 months)	2.6±1.7	4.4±2.0	4.7±1.6	5.663	0.059	
LEQ—sum of life events (>6 months ago)	2.2±2.2	4.2±3.5	4.1±2.0	3.643	0.162	

Abbreviations: BDI, Beck Depression Inventory; BIS/BAS, behavioural inhibition system/behavioural activation system; EPQRS, Eysenck Personality Questionnaire; K10, Kessler Psychological Distress Scale; LEQ, Life Events Questionnaire; MA, methamphetamine-dependent subjects with no psychotic events; MAP, methamphetamine-associated psychosis; PANSS, Positive and Negative Syndrome Scale.

Shapiro wilk test was used to assess normality of variables and either a one-way analysis of variance (ANOVA) or KRUSKAL–Wallis ANOVA with *post hoc* Bonferroni correction was implemented accordingly.

**Table 2:** Brain structural volumes (mm^3^) from all the subjects (*N*=30)

*Brain region*	*Healthy controls* *(*N*=10)*	*MA* *(*N*=10)*	*MAP* *(*N*=10)*	*Bayes* *ANOVA*		Post hoc *significance*
	*Mean±s.d.*	*Mean±s.d.*	*Mean±s.d.*	X^*2*^*(df=2)*	P*-value*	*Bonferroni* P-value
L hippocampus	3950.11±463.71	3790±297.51	3521.71±173.43	3.538	0.041	Control > MAP
R hippocampus	4067.56±414.08	4005.43±196.29	3645.29±189.97	4.261	0.029	Control > MAP
L accumbens	690.56±80.38	689.14±128.15	651.57±99.24	0.343	0.714	
R accumbens	669.33±100.54	673.00±199.23	694.71±91.48	0.076	0.927	
L caudate	4116.89±340.84	4078.57±293.78	3906.71±177.23	1.149	0.337	
R caudate	4211.22±251.11	4283.86±314.36	4119±163.64	0.760	0.481	
L putamen	6606.78±408.97	6633.14±667.17	6718.57±661.5	0.078	0.925	
R putamen	6313.33±371.03	6274.43±596.45	6506.71±672.14	0.373	0.694	
L ventral DC	4551.33±247.16	4295.71±273.56	4323.71±204.25	2.715	0.091	
R ventral DC	4473.44±377.34	4340.43±78.7	4369.86±278.58	0.485	0.623	
CC anterior	938.78±125.96	1056.14±194.83	1016.57±100.31	1.389	0.272	
CC posterior	966.00±191.65	912.29±139.86	956.29±135.16	0.236	0.792	

Abbreviations: CC, corpus callosum; DC, diencephalon; L, left; R, right.

Bayes analysis of variance (ANOVA) parameters: conf=12, Bayes=1, winSize=5. *P*-values corrected for multiple comparisons.

**Table 3:** Top informative features for separating controls from METH subjects (25 genes) and MA from MAP subjects (20 genes)

*Gene symbol*	*Parametric* P*-value*	*%CV support*	*Module correspondence*	*Significant positive module-trait correlations*	*Significant negative module-trait correlations*
***Top 25 informative features separating controls from METH subjects***					
***ELK3***^†^	0.0175377	97	Circadian clock	EPQRS psychoticism (*r*=0.43, *P*=0.02) CC posterior (*r*=0.39, *P*=0.03)	EPQRS extraversion (*r*=−0.38, *P*=0.04)
					
*CRTAM*	0.03485	97	Generic transcription	EPQRS neuroticisim (*r*=0.41, *P*=0.02) EPQRS total (*r*=0.37, *P*=0.05)	Left accumbens (*r*=−0.49, *P*=0.0006) Right accumbens (*r* =−0.6, *P*=0.00005)
*** MAGEE1***	0.0158379	100	Generic transcription		
					
***RNF138P1***	0.0078459	87	RNA degradation	Control status (*r*=0.38, *P*=0.04) Left ventral DC (*r*=0.37, *P*=0.04)	CC anterior (*r*=−0.48, *P*=0.008) Right accumbens (*r*=−0.5, *P*=0.005)
*MFN1*	0.0070206	87	RNA degradation		
TBC1D2*	0.000805	90	RNA degradation		
* ZNF286B*	0.000065	90	RNA degradation		
*MRPL50*	0.0001267	93	RNA degradation		
* ADAM15* ^†^	0.000731	97	RNA degradation		
*** DDRGK1***	0.0055266	97	RNA degradation		
*** MTHFSD***	0.0612426	97	RNA degradation		
***ARL6***	0.0037554	97	RNA degradation		
*GKAP1*	0.0009407	97	RNA degradation		
*** FAM169A***	0.0008839	97	RNA degradation		
*KBTBD6*	0.0003548	97	RNA degradation		
*** ZSCAN5A***	0.0012892	100	RNA degradation		
***FBN1***^†,^*	0.0168567	100	RNA degradation		
***ZNF821***	0.0193724	100	RNA degradation		
***FBP1***^†^	0.6900462	100	RNA degradation		
*CDK7*	0.0054503	100	RNA degradation		
					
*CDYL2*	0.0000834	93	RNA-binding	K10 total (*r*=0.42, *P*=0.02)	Right ventral DC (*r*=−0.42, *P*=0.02)
*TOMM34*	0.0019291	100	RNA-binding		
					
***C7orf11***	0.0587445	80	Ubiquitin-mediated proteolysis	Control status (*r*=0.4, *P*=0.03) Left caudate (*r*=0.37, *P*=0.04) Left ventral DC (*r*=0.48, *P*=0.0007)	MAP status (*r*=−0.45, *P*=0.01) K10 Total (*r*=−0.43, *P*=0.02) CC Anterior (*r*=−0.55, *P*=0.002) Right Accumbens (*r*=−0.4, *P*=0.03)
*UHMK1*^†^	0.6057577	97	Ubiquitin-mediated proteolysis		
*** PHLDB2***	0.0007613	100	Ubiquitin-mediated proteolysis		

***Top 20 informative features separating MA from MAP subjects***					
SIN3A*	0.0926295	70	Circadian clock	EPQRS psychoticism (*r*=0.43, *P*=0.02) CC posterior (*r*=0.39, *P*=0.03)	EPQRS extraversion (*r*=−0.38, *P*=0.04)
***ELK3***^†^	0.0002902	90	Circadian clock		
					
*** MAGEE1***	0.0001558	100	Generic transcription	EPQRS neuroticisim (*r*=0.41, *P*=0.02) EPQRS total (*r*=0.37, *P*=0.05)	Left accumbens (*r*=−0.49, *P*=0.0006) Right accumbens (*r*=−0.6, *P*=0.00005)
					
*MFSD7*	0.0440767	85	Interferon signalling	MA dep. status (*r*=0.4, *P*=0.03) BDI total (*r*=0.4, *P*=0.03) Left putamen (*r*=0.54, *P*=0.002) Right putamen (*r*=0.41, *P*=0.03)	EPQRS extraversion (*r*=−0.43, *P*=0.02) EPQRS total (*r*=−0.43, *P*=0.02) CC posterior (*r*=−0.43, *P*=0.02)
					
* SLC41A3*	0.0018933	100	Ribosome pathway	EPQRS total (*r*=0.38, *P*=0.04) BDI total (*r*=0.44, *P*=0.02)	MA status (*r*=−0.37, *P*=0.04)
					
*** MTHFSD***	0.0002405	90	RNA degradation	Control status (*r*=0.38, *P*=0.04) Left ventral DC (*r*=0.37, *P*=0.04)	CC anterior (*r*=−0.48, *P*=0.008) Right accumbens (*r*=−0.5, *P*=0.005)
ZNF821*	0.11798	90	RNA degradation		
FBP1*	0.3549855	90	RNA degradation		
*** RNF138P1***	0.0195014	90	RNA degradation		
***ARL6***	0.0317958	95	RNA degradation		
*ETFA*	0.0235683	95	RNA degradation		
TBC1D2*	0.157939	100	RNA degradation		
*** FAM169A***	0.0132909	100	RNA degradation		
*** ZSCAN5A***	0.0112376	100	RNA degradation		
*CLN3*	0.0087815	100	RNA degradation		
*** DDRGK1***	0.0082958	100	RNA degradation		
***FBN1***^†,^*	0.0070075	100	RNA degradation		
					
*PIGF*	0.1818898	90	Ubiquitin-mediated proteolysis	Control status (*r*=0.4, *P*=0.03) Left caudate (*r*=0.37, *P*=0.04) Left ventral DC (*r*=0.48, *P*=0.0007)	MAP status (*r*=−0.45, *P*=0.01) K10 Total (*r*=−0.43, *P*=0.02) CC Anterior (*r*=−0.55, *P*=0.002) Right Accumbens (*r*=−0.4, *P*=0.03)
***C7orf11***	0.0028377	90	Ubiquitin-mediated proteolysis		
***PHLDB2***	0.1302545	95	Ubiquitin-mediated proteolysis		

Abbreviations: BDI, Beck Depression Inventory; CC, corpus callosum; DC, diencephalon; EPQRS, Eysenck Personality Questionnaire.

Parametric *P*-value indicates significance in a strict sense following 1000 random permutations to group labels using small *N*. %CV support denotes the number of correctly passed cross-validations for each gene. Module correspondence is the module membership of each gene and the subsequent significant correlations for each module are depicted. Genes in bold are those that were used in classification for nodes 1 and 2 (14 genes total). (*) indicates genes found dysregulated in the blood of psychosis studies; (^†^) indicates genes found as genetic variants in SCZ studies.
