# Supplementary figures and images for: Candidate gene networks and blood biomarkers of methamphetamine-associated psychosis: an integrative RNA-sequencing report
Source: Transl Psychiatry. 2016 May 10;6(5):e802–. doi: 10.1038/tp.2016.67 (PMC5070070; doi:10.1038/tp.2016.67)

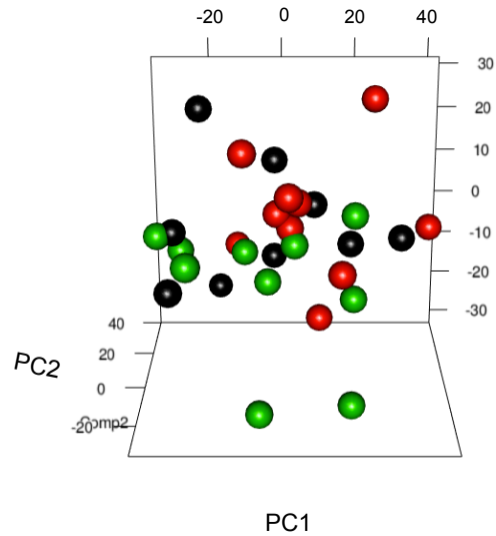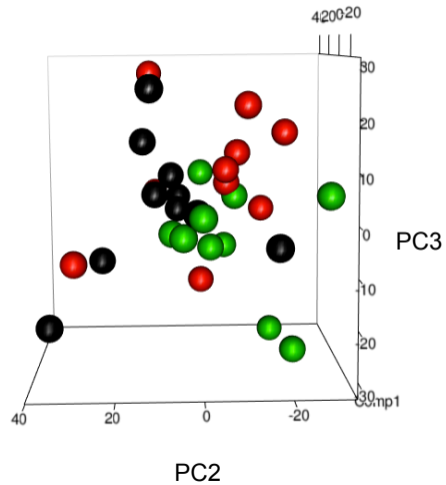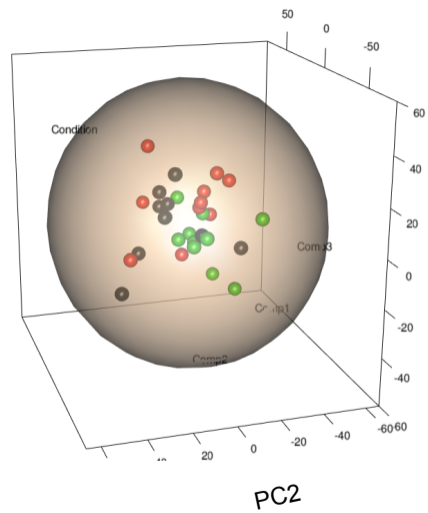

Supplement: Supplementary Figure 1 [file tp201667x1.pdf]

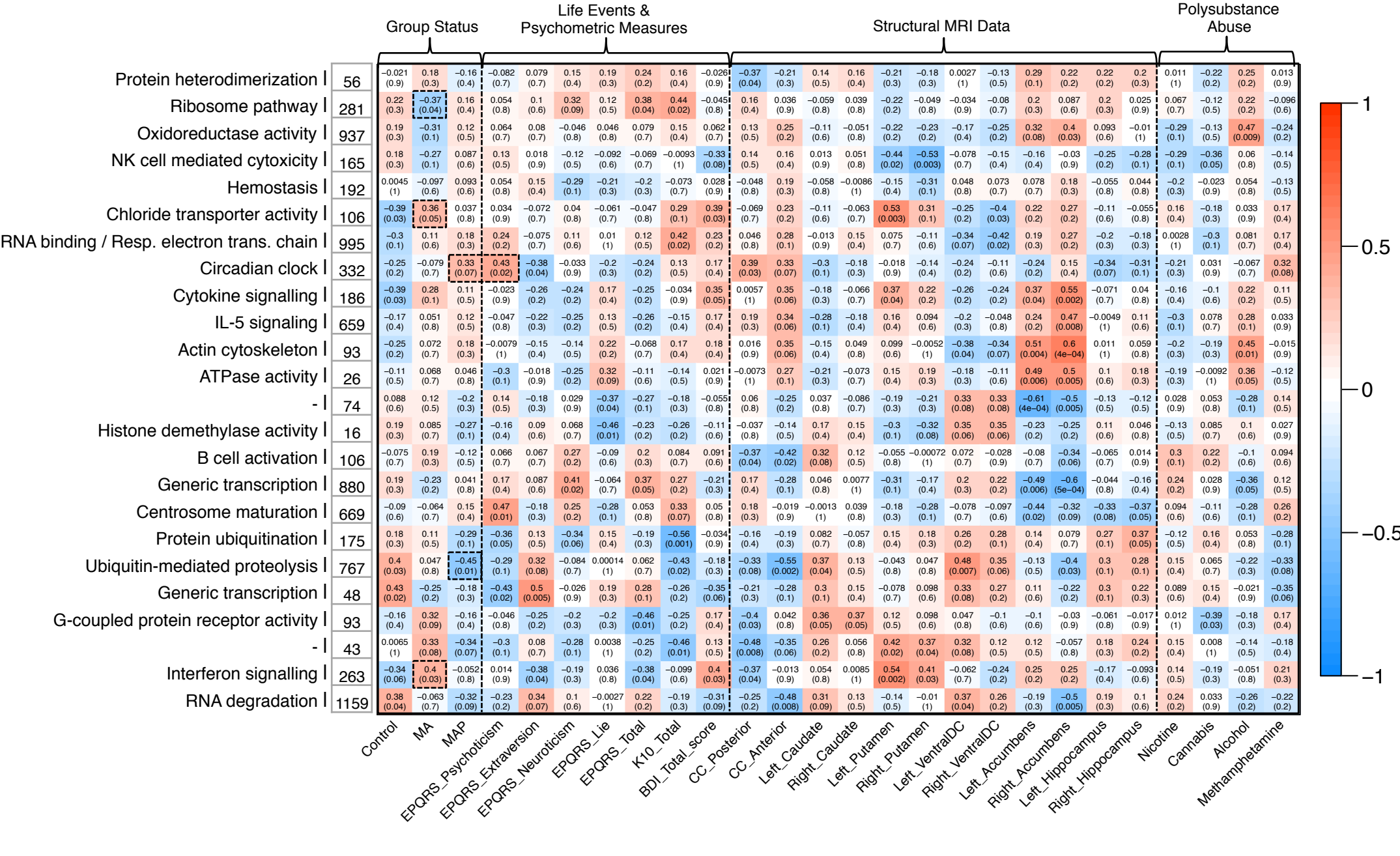

Supplement: Supplementary Figure 3 [file tp201667x3.pdf]

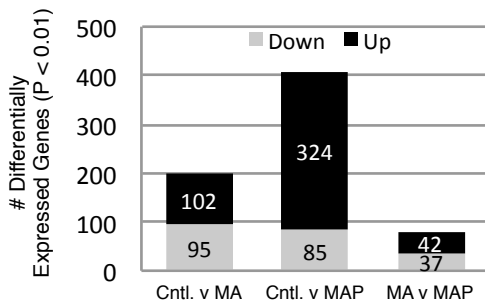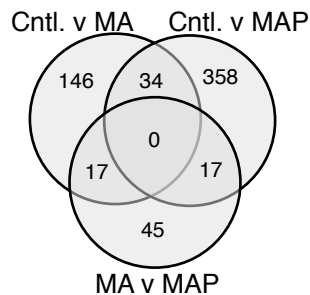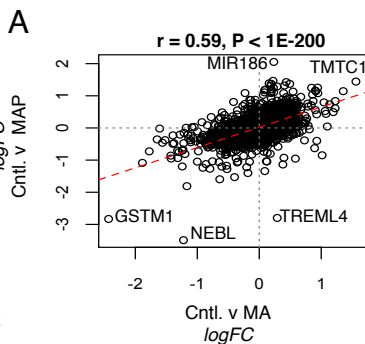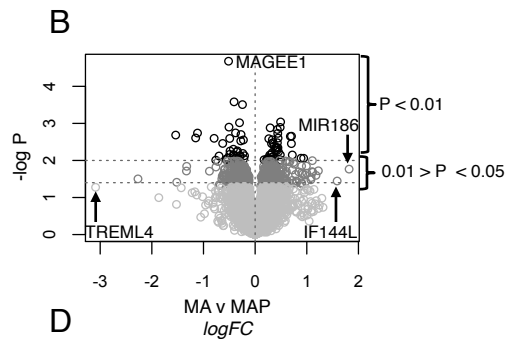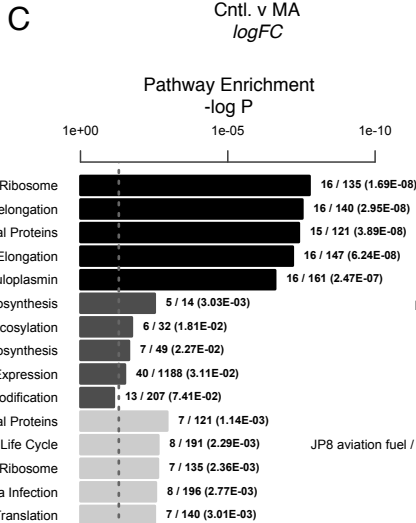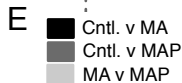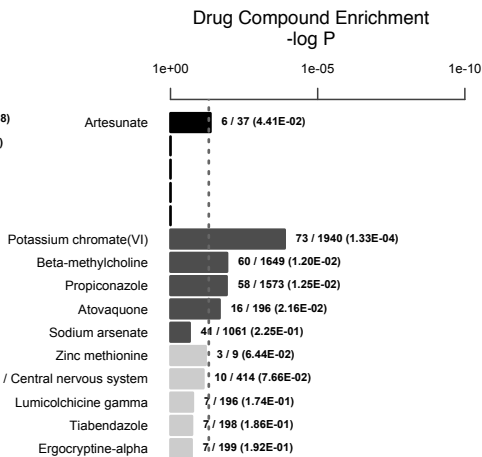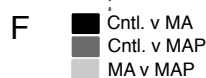

Supplement: Supplementary Figure 4 [file tp201667x4.pdf]
