# Supplementary material for: Candidate gene networks and blood biomarkers of methamphetamine-associated psychosis: an integrative RNA-sequencing report
Source: Transl Psychiatry. 2016 May 10;6(5):e802–. doi: 10.1038/tp.2016.67 (PMC5070070; doi:10.1038/tp.2016.67)
Supplement: Supplementary Figure 2 [file tp201667x2.pdf]

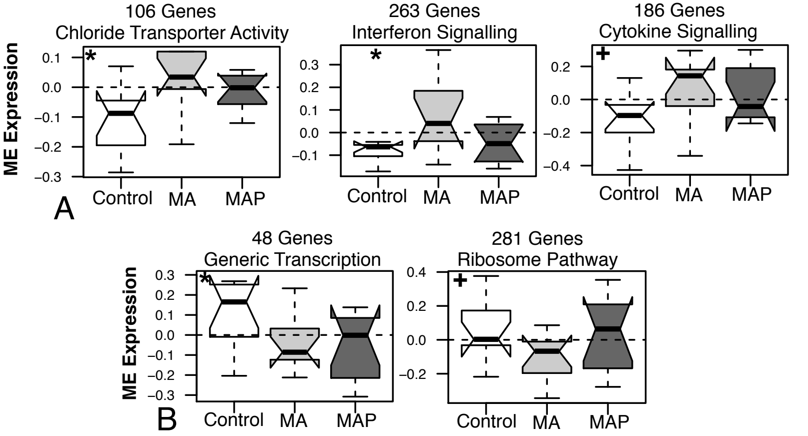
106 Genes
Chloride Transporter Activity
263 Genes
Interferon Signalling
186 Genes
Cytokine Signalling
ME Expression
0.1
0.0
−0.1
−0.2
−0.3
0.3
0.2
0.4
−0.4
Control
MA
MAP
A
48 Genes
Generic Transcription
281 Genes
Ribosome Pathway
B
