# Supplementary material for: Candidate gene networks and blood biomarkers of methamphetamine-associated psychosis: an integrative RNA-sequencing report
Source: Transl Psychiatry. 2016 May 10;6(5):e802–. doi: 10.1038/tp.2016.67 (PMC5070070; doi:10.1038/tp.2016.67)
Supplement: Supplementary Figure 5 [file tp201667x5.pdf]

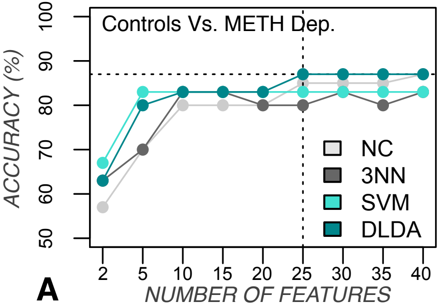

A

Top 25 performing features

	NC	3NN	SVM	DLDA
P-value *	< 0.001	0.01	0.012	< 0.001
Sensitivity	0.80	0.50	0.60	0.80
Specificity	0.95	0.95	0.95	0.90
AUC	0.85	0.80	0.83	0.87
95% CI	0.74-0.96	0.76-0.94	0.70-0.96	0.75-0.99

B

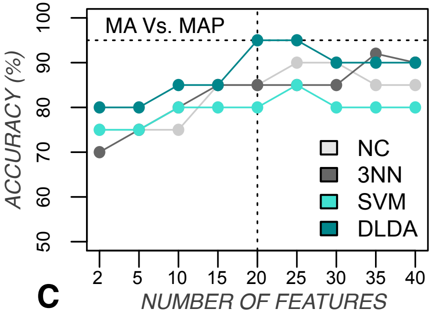

C

Top 20 performing features

	NC	3NN	SVM	DLDA
P-value *	0.005	0.003	0.02	0.001
Sensitivity	0.80	0.90	0.90	0.90
Specificity	0.90	0.80	0.70	1
AUC	0.85	0.85	0.80	0.95
95% CI	0.74-0.99	0.74-0.99	0.72-0.98	0.84-0.99

D
