# Supplementary material for: Candidate gene networks and blood biomarkers of methamphetamine-associated psychosis: an integrative RNA-sequencing report
Source: Transl Psychiatry. 2016 May 10;6(5):e802–. doi: 10.1038/tp.2016.67 (PMC5070070; doi:10.1038/tp.2016.67)
Supplement: Supplementary Figure 6 [file tp201667x6.pdf]

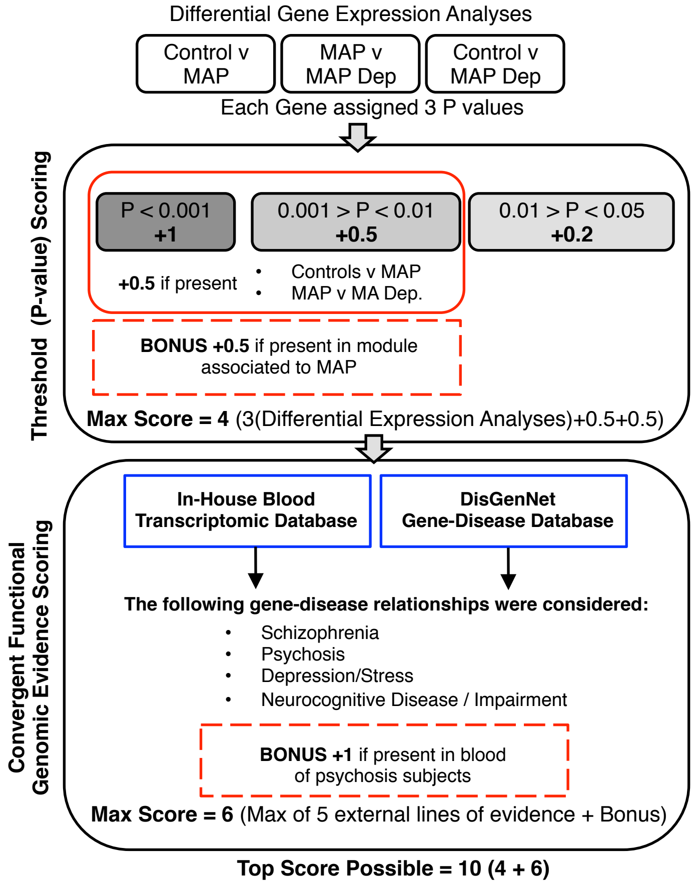
Differential Gene Expression Analyses
Control v MAP
MAP v MAP Dep
Control v MAP Dep
Each Gene assigned 3 P values
Threshold (P-value) Scoring
P < 0.001 +1
0.001 > P < 0.01 +0.5
0.01 > P < 0.05 +0.2
+0.5 if present
Controls v MAP
MAP v MA Dep.
BONUS +0.5 if present in module associated to MAP
Max Score = 4 (3(Differential Expression Analyses)+0.5+0.5)
Convergent Functional Genomic Evidence Scoring
In-House Blood Transcriptomic Database
DisGenNet Gene-Disease Database
The following gene-disease relationships were considered:
Schizophrenia
Psychosis
Depression/Stress
Neurocognitive Disease / Impairment
BONUS +1 if present in blood of psychosis subjects
Max Score = 6 (Max of 5 external lines of evidence + Bonus)
Top Score Possible = 10 (4 + 6)
