# Supplementary material for: Candidate gene networks and blood biomarkers of methamphetamine-associated psychosis: an integrative RNA-sequencing report
Source: Transl Psychiatry. 2016 May 10;6(5):e802–. doi: 10.1038/tp.2016.67 (PMC5070070; doi:10.1038/tp.2016.67)
Supplement: Supplementary Figure Legends [file tp201667x7.docx]

**Main Figure Legends**

**Figure 1.** A multi-step translational work-flow for identifying MAP biomarkers. First, WGCNA analysis built a global co-expression network and identified 24 co-expression modules. On the hierarchical cluster tree each line represents a gene (leaf) and each group of lines represents a discrete group of co-regulated genes, or gene modules (branch) on the clustering gene tree. Each gene module is indicated by the colour bar below the dendrogram, and subsequently functionally annotated then integrated with recorded clinical and biological data to identify candidate gene modules representing functional biomarkers of MAP. Second, differential gene expression and class prediction methods identified 20 candidate MAP biomarkers (14 were recycled from the second split on the tree). A Bayesian-like convergent functional genomic (CFG) approach prioritized our panel of biomarkers specific to MAP and biomarkers were placed within an empirically derived biological framework. For each step the corresponding figure and/or table is listed providing a quick reference.

**Figure 2.** Significant MAP findings from differential analysis of module eigengene (ME) values and brains structural volumes (mm3) across controls (white), MA subjects (light grey) and MAP subjects (dark grey). Modules specific to MAP include **(a)** ubiquitin(UB)-mediated proteolysis, **(b)** RNA degradation and **(c)** circadian clock. Indicated for each module are, number of overlapping genes from the module ∩ out of total genes in the term. Enrichment *P* values are Bonferroni corrected for multiple comparisons. A Bayes ANOVA (parameters: conf=12, bayes=1, winSize=5) was used on ME values to test for significance between groups and *P* values were corrected multiple comparisons where (*) implies post-hoc corrected *p*-value significance < 0.05 and (^+^) indicates *p*-value significance < 0.05 without post-hoc correction.

**Figure 3.** Top candidate blood biomarkers for MAP. (**a**) CFG evidence and scoring are depicted on the right side of the pyramid. Genes in bold have been found in external publications. Genes found in METH-free studies investigating SCZ (^†^) and psychosis (*) are as indicated. (**b**) Overlapping gene-disease relationships including CFG validated genes within gene modules (ubiquitin-mediated proteolysis & circadian rhythm) and single gene biomarkers. Nodes represent genes and edges indicate gene-disease relationships. Node shape denotes empirically derived functions from our network analysis. Green shading indicates biomarkers from our machine-learning analysis including 14 unique genes separating controls from METH dependants. Grey nodes represent CFG validated biomarkers of delusion (psychosis) or SCZ^(11,17)^. Node border colour in turquoise indicates gene signatures across MAP, general psychosis and SCZ studies. Venn diagram depicts lack of overlap from curated haloperidol-gene signatures onto the 128 candidate MAP genes (61 UPS + 39 clock + 25 + 20 =128 genes (while accounting for overlap across lists)).

**Supplementary Figure Legends**

**Supplementary Figure 1.** Principal component analysis (PCA) for quality control of normalized RNA-Seq data. A total of 12,128 genes were using the first three principal components for MAP (green), MA (red) and control subjects (black) and visualized using three distinct advantage points. PCA plot on the far right measures 2 standard deviations from the average for detecting putative outliers.

**Supplementary Figure 2.** Module eigengene (ME) differential expression analysis across controls (white), MA dependent subjects (light grey) and MAP subjects (dark grey). (**A**) ME expression values over-expressed in MA subjects relative to controls include modules enriched for chloride transporter activity, interferon signalling and cytokine signalling. (**B**) ME expression values under-expressed in MA subjects relative to controls include modules enriched for generic transcription and ribosome pathway. A Bayes ANOVA (parameters: conf=12, bayes=1, winSize=5) was used on ME values to test for significance between groups and corrected for multiple comparisons where (*) implies post-hoc corrected *p*-value significance < 0.05 and (^+^) indicates *p*-value significance < 0.05 without post-hoc correction.

**Supplementary Figure 3.** Module eigengene (ME) associations. Functional gene modules were associated with external data including self-reported measurements, life history, behavioural and depression scores, structural MRI data and polysubstance abuse (confounding factors). The primary function of each gene co-expression module is labelled on the y-axis with the corresponding number of genes with kME > 0.5 within each module. The measure of correlation, *r*, is the top value in each box and the related *p*-value is designated below within brackets. *P* values < 0.002 pass the most conservative multiple comparison correction (Bonferroni). Red signifies a positive correlation and blue signifies a negative correlation as indicated by colour scale. Significant MA and MAP associations are outlined in boxes. Due to the high number of self-reported measures and structural MRI data we only report on those variables that drew at least one significant ME association. ‘-‘ indicates no principal function was identified.

**Supplementary Figure 4.** Differential gene expression analyses. (**A**) The total number of over- and under-expressed genes (*P* < 0.01) are shown for each pair-wise group comparison and subsequently (**B**) the overlap of all identified genes are displayed. (**C**) Log fold-change (logFC) of all genes between controls and MAP subjects were associated with logFC values for genes between controls and MA subjects, MAP or MA specific genes are labelled. (**D**) Volcano plot (logFC vs. log *p*-value) of dysregulated genes between MA and MAP subjects, *P* values coloured by significance. (**E**) The top 5 most significantly enriched pathways and (**F**) drug-compounds for each pair-wise comparison (Bonferroni *p* < 0.05).

**Supplementary Figure 5.** Two separate models were used to predict group outcomes. (**A**) Gene expression classifier accuracies achieved when discriminating between healthy control and METH dependent subjects (MA + MAP groups) and (**B**) results of the top performing model containing 25 genes are displayed. (**C**) Gene expression classifier accuracies achieved when discriminating between MA subjects from MAP subjects and (**D**) results of the top performing model containing 20 genes are displayed. In each case supervised class prediction was performed using different combinations of genes with Recursive Feature Elimination and evaluated with four different multivariate classification methods. Abbreviations; P-value *, result of 1000 random permutations to class labels; AUC, overall balanced accuracy; CI, confidence interval; NC, nearest centroid; 3NN, three-nearest neighbors; SVM, support vector machine; DLDA, diagonal linear discriminate analysis.

**Supplementary Figure 6.** Convergent Functional Genomic (CFG) scoring scheme. First, each gene received a score based on *p*-value threshold. A score of 1 was given for *P* < 0.001, a score of 0.5 for 0.001 > *P* < 0.01 and a score of 0.2 for 0.01 > *P* < 0.05. A gene was given an additional score of 0.5 if *P* < 0.01 between MAP and controls subjects as well as MAP and MA subjects. A bonus 0.5 point was given if this gene was found in a functional module associated to MAP or psychosis (i.e. ubiquitin-mediated proteolysis or circadian rhythm modules). Thus, the maximum score based on this first series of thresholds is 4 (3(differential expression analyses) + 0.5 + 0.5). Second, we used CFG evidence as identified from two databases; (i) an in-house blood transcriptomic database and (ii) DisGenNet database. We only used gene-disease relationships for the following diseases: schizophrenia, psychosis, depression/stress and neurocognitive impairment. A maximum of 5 external lines of evidence were allowed. A bonus point of 1 was granted if present in the blood of previous psychosis studies. Thus, the maximum score attainable is 6 (5 lines of evidence + 1) and the top score possible for each gene considering all possible combinations of points is 10 (4 + 6).
